# Magnetism Induced by Azanide and Ammonia Adsorption in Defective Molybdenum Disulfide and Diselenide: A First-Principles Study

**DOI:** 10.1021/acsomega.5c10979

**Published:** 2026-01-20

**Authors:** Guilherme S. L. Fabris, Bruno Ipaves, Raphael B. Oliveira, Humberto R. Gutierrez, Marcelo L. Pereira Junior, Douglas S. Galvão

**Affiliations:** † Applied Physics Department and Center for Computational Engineering & Sciences, 28132 State University of Campinas, Campinas, São Paulo 13083-970, Brazil; ‡ Department of Materials Science and NanoEngineering, 3990 Rice University, Houston, Texas 77005, United States; § Department of Physics, 7831 University of South Florida, Tampa, Florida 33620, United States; ∥ Department of Electrical Engineering, College of Technology, University of Brasília, Brasília, Federal District 70910-900, Brazil

## Abstract

Two-dimensional (2D) transition metal dichalcogenides (TMDs) have attracted considerable attention due to their tunable structural, electronic, and spin-related properties, particularly in the presence of point defects and molecular adsorbates. Motivated by these aspects, we have investigated using first-principles methods, the magnetic properties induced by azanide (NH_2_) and ammonia (NH_3_) adsorption on defective monolayers of molybdenum disulfide (MoS_2_) and molybdenum diselenide (MoSe_2_). Spin-polarized density functional theory (DFT) at the generalized gradient approximation (GGA) level, using the Perdew–Burke–Ernzerhof (PBE) functional, was employed to investigate the impact of mono- and divacancies on the local spin environment and the role of molecular adsorption in modifying magnetic behavior. The results show that pristine chalcogen vacancies do not generate magnetism, whereas the adsorption of NH_2_ and NH_3_ creates localized magnetic moments in Mo-based dichalcogenides. A notable case occurs for MoSe_2_, where NH_3_ dissociation into NH_2_ and H fragments on the same side of the surface produces a net magnetic moment of 2.0 μ_B_. Tests performed on W-based dichalcogenides under equivalent conditions showed no magnetic response and are reported here only for comparison. These findings demonstrate that molecular adsorption combined with defect engineering can be a practical approach to tune magnetism in 2D materials, with potential relevance for spintronic and sensing applications.

## Graphical abstract

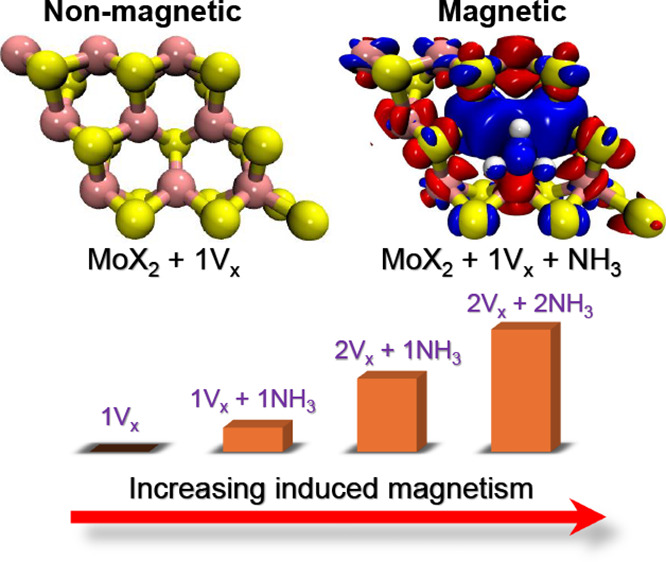

## Introduction

Two-dimensional (2D) transition metal dichalcogenides (TMDs) have attracted considerable attention due to their tunable electronic,^1,2^ optical,^3,4^ and structural^5,6^ properties, which make them relevant for diverse technological applications.^6^ Beyond their intrinsic characteristics, the behavior of TMD monolayers can be significantly modified by the presence of point defects, such as mono and divacancies, as well as by the adsorption of gas molecules.^7–11^ Previous studies indicate that vacancies of chalcogen atoms (S or Se) typically do not induce spin density variations in Mo-based dichalcogenides. In contrast, metal vacancies can give rise to localized magnetic moments.^12–14^

The role of molecular adsorption in defective TMDs has been examined for species such as H_2_O, O_2_, and O_3_, which can stabilize near vacancy sites and, in some cases, dissociate to alter the local electronic structure.^8,15^ In parallel, defects such as vacancies and interstitials, which are inevitably formed during material growth, may generate local magnetic moments capable of interacting over long ranges.^13^ These findings underline the importance of modifications in the spin density environment and suggest that the impact of adsorbates depends strongly on the type of vacancy and the surrounding chemical environment.

While several studies have examined adsorption^16,17^ and defect^18,19^ effects in TMDs, systematic investigations specifically addressing small adsorbates such as NH_2_ and NH_3_ on Mo-based TMDs remain underexplored. Moreover, the combined influence of mono- and divacancies together with multiple adsorbates, and its impact on the spatial distribution of local magnetic moments, has not been comprehensively explored. Addressing this knowledge gap is important for understanding spin distributions in 2D TMDs and for advancing the design of sensor and spintronic applications.^20,21^

In this work, we have carried out first-principles density functional theory (DFT) simulations to investigate the effects of azanide (NH_2_) and ammonia (NH_3_) adsorption on monolayers of MoS_2_ and MoSe_2_ containing mono- and divacancies. We analyzed how adsorbates modify the local spin densities and identified conditions under which magnetism can be induced in otherwise nonmagnetic defective chalcogens. For comparison, additional tests on WS_2_ and WSe_2_ showed no magnetic response under equivalent conditions. These results provide insights into adsorbate-driven spin distortions in Mo-based dichalcogenides and contribute to a better understanding of defect-engineered 2D materials with tunable spin properties.

## Methodology

To investigate the structural, electronic, and spin densities of MoX_2_ (X = S or Se) with mono and divacancies, as well as the effect of ammonia and azanide molecules on their magnetic properties, we performed ab initio simulations based on DFT^22^ as implemented in the SIESTA code.^23,24^ The exchange-correlation effects were described using the PBE (Perdew–Burke–Ernzerhof) functional,^25^ combined with a DZP (double-ζ polarization) basis set composed of numerical atomic orbitals. This choice follows the widespread use of PBE in studies of defect states and adsorption effects in Mo-based TMDs.^12,15,26,27^ A real-space mesh cutoff of 400 Ry and a Γ-centered Monkhorst–Pack grid^28^ of 8 × 8 × 1 and 4 × 4 × 1 *k*-points were adopted for the monolayer unit cell and the 3 × 3 × 1 supercell, respectively. A vacuum region of 25 Å was included along the perpendicular direction to avoid spurious interactions between periodic images. The 3 × 3 × 1 supercell provides defect-image separations larger than 9 Å for MoS_2_ and MoSe_2_, ensuring a reliable description of isolated vacancies and of the NH_2_/NH_3_ adsorption configurations considered. The convergence threshold for the self-consistent field cycle was set to 10^–4^ for the density matrix tolerance, and the structural relaxation was performed until the residual forces were smaller than 0.05 eV/Å. All calculations were carried out within a spin-polarized framework. The nanostructures and the spin density were visualized using the Visual Molecular Dynamics (VMD) software,^29^ and an isovalue of 0.001 was adopted for the spin-density plots.

## Results

Initially, we investigated the changes in the spin densities of MoS_2_ and MoSe_2_ monolayers in the presence of defects. As a first step, we optimized the pristine MoX_2_ structures to validate the accuracy of the computational setup. The optimized lattice parameters were *a* = *b* = 3.17 Å for MoS_2_ and *a* = *b* = 3.31 Å for MoSe_2_, with corresponding Mo–S and Mo–Se bond lengths of 2.42 and 2.54 Å. The calculated electronic band gaps of pristine MoS_2_ and MoSe_2_ are 1.14 and 1.08 eV, respectively. These values are consistent with previous theoretical reports,^7,8,26,27^ as expected given the well-known tendency of PBE-based calculations to underestimate band gaps. The results are summarized in Table 1, confirming the reliability of the adopted methodology.

**Table 1:** Comparison of the In-plane Lattice Constant *a* (Å) and Electronic Band Gap *E*_g_ (eV) of Monolayer MoX_2_ Obtained in This Work, Together With Representative Hybrid-functional and Experimental Values

	*a* (Å)			*E*_g_ (eV)		
material	this work	hybrid^30^	expt.^31,32^	this work	hybrid^30^	expt.^31,32^
MoS_2_	3.17	3.23	3.16	1.14	1.56	1.80
MoSe_2_	3.31	3.38	3.30	1.08	1.38	1.54–1.57

After validating the computational parameters, we created a 3 × 3 × 1 supercell of MoX_2_ (X = S or Se) and introduced mono- and divacancy models. The divacancy was considered in two configurations: both vacancies on the same side of the monolayer and one vacancy on each side of the supercell (alternated), as illustrated in Figure 1. These defective models were then used to investigate the influence of NH_3_ adsorption at the vacancy sites, with representative configurations shown in Figure 1.

**Figure 1:**
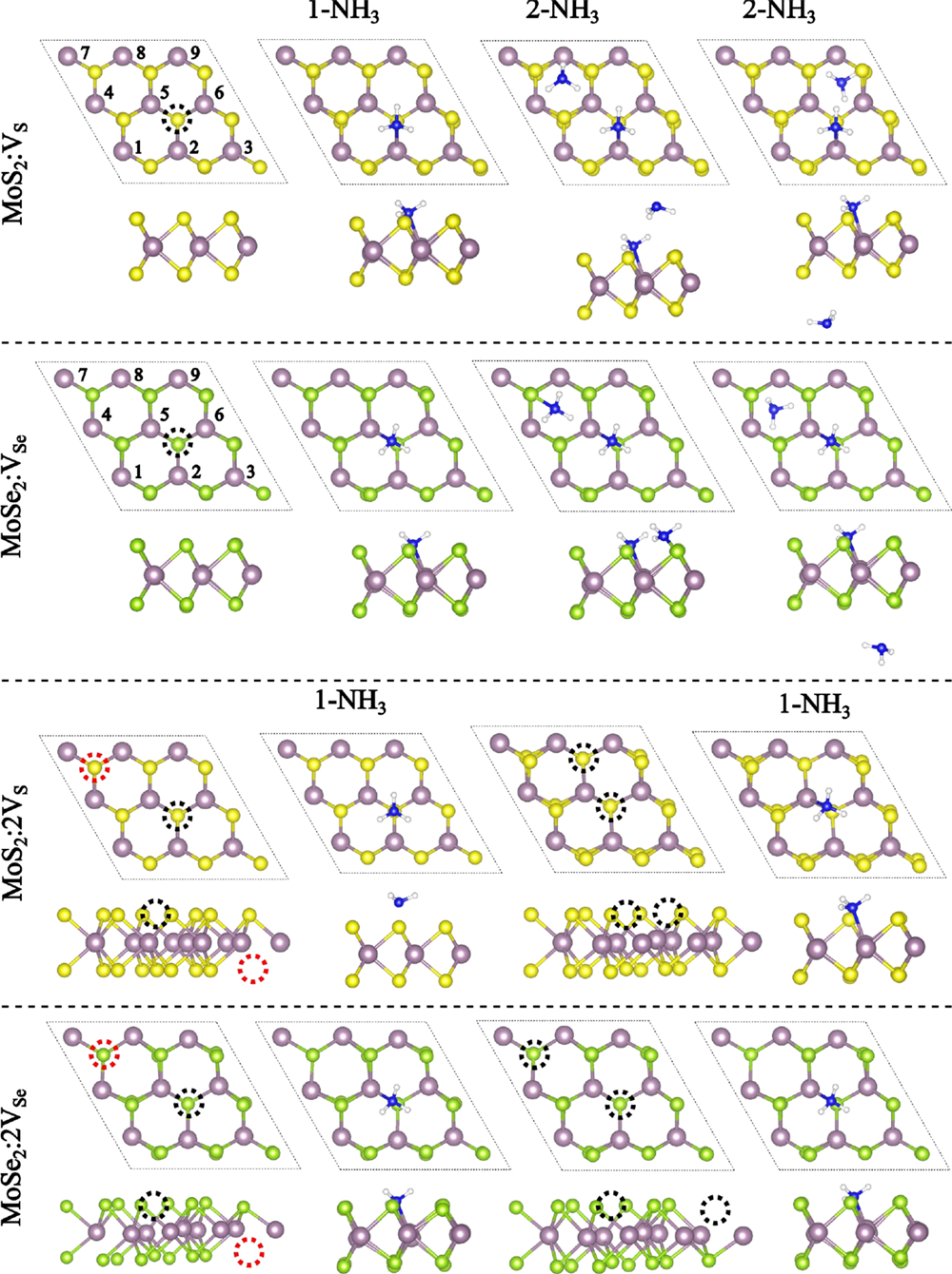
Schematic illustration of the top and side views of a 3 × 3 × 1 supercell of MoX_2_ (X = S or Se) featuring one vacancy (VX) and two vacancies (2VX). The figure includes cases investigated with the adsorption of one NH_3_ molecule and two NH_3_ molecules. Dashed black and red circles indicate the vacancy positions, while the numbers 1–9 correspond to the label of the Mo atoms referenced in all tables.

Before analyzing the adsorption results, it is important to note that the dangling bonds associated with chalcogen vacancies are explicitly present in our defective MoX_2_ models. Removing an S or Se atom leaves the surrounding Mo atoms unsaturated, generating localized states at the vacancy site; these vacancy-induced dangling bonds interact directly with the adsorbed NH_2_/NH_3_ species and play an essential role in the magnetic behavior discussed below. All structures discussed in this work were fully relaxed before evaluating their magnetic properties, and the analysis focuses on the response associated with NH_2_ and NH_3_ molecules near vacancy sites. The adsorption of azanide or ammonia on MoX_2_ generally occurs via physisorption, which may lead to slight variations in the electronic band gap value due to polarization effects. However, they do not introduce midgap states.^33^

The creation of S and Se vacancies resulted in defective structures without spin density variations, in agreement with previous reports that describe the absence of magnetism in such systems.^7,8,26,27^ The adsorption of H_2_O and NH_3_ in Mo-based TMDs has been examined in the literature, and most studies indicate negligible changes in the spin environment.^8,9,34^ Nevertheless, the dissociation of H_2_O at vacancy sites has been reported to induce significant modifications in the local spin distribution.^8,15^

For the NH_3_ adsorption, a single molecule stabilizes near the vacancy site at distances of 2.38 and 2.36 Å from the nearest Mo atom in MoS_2_ and MoSe_2_, respectively. When two NH_3_ molecules are present, one occupies a position similar to that in the single-molecule case. At the same time, the second stabilizes further from the surface, with distances to the nearest S or Se atoms ranging from 1.79 to 2.94 Å.

A key result of this study is that NH_3_ adsorption increases the local magnetic moment of Mo atoms near vacancy sites in both MoS_2_ and MoSe_2_ (Figures 2 and 3, Tables 2 and 3). This effect, rarely reported in the literature, was observed at specific Mo atoms surrounding the adsorption site. In the case of a divacancy on the same side of the monolayer, the increase became more pronounced, with additional Mo atoms developing nonzero magnetic moments. Quantitatively, MoS_2_ exhibited an enhancement of approximately 21.3%, while MoSe_2_ showed a much larger increase of 200%. By contrast, the alternated divacancy configuration did not modify the response, yielding values comparable to those of the single-vacancy case. These results indicate that exposure to NH_3_ can enhance induced magnetism in Mo-based dichalcogenides, as illustrated by the spin density difference maps in Figures 2 and 3.

**Figure 2:**
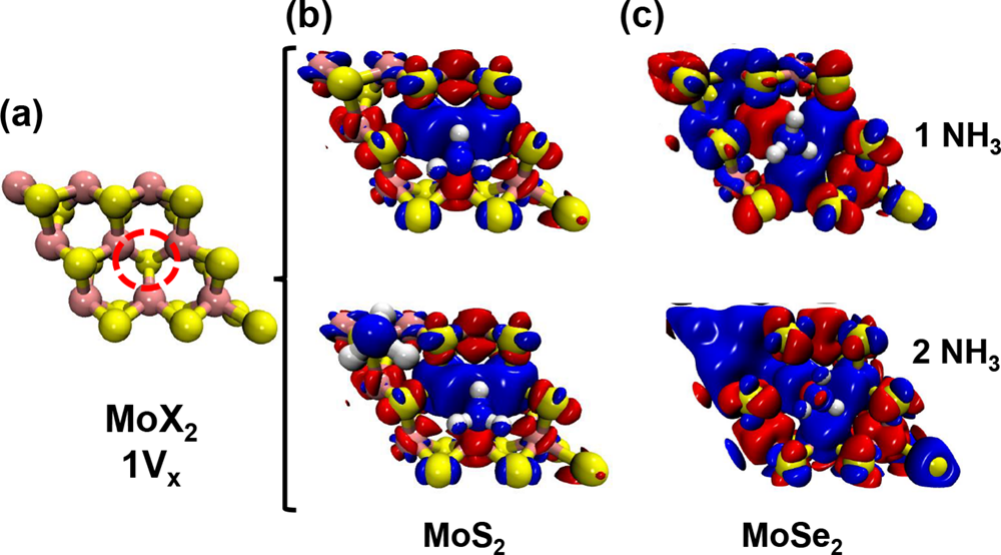
Spin density maps for (a) MoX_2_ monolayers with a monovacancy, (b) MoS_2_, and (c) MoSe_2_ with one and two adsorbed NH_3_ molecules. The dashed red circle indicates the position of the vacancy. In the maps, red color indicates spin-up density, while blue represents spin-down density.

**Figure 3:**
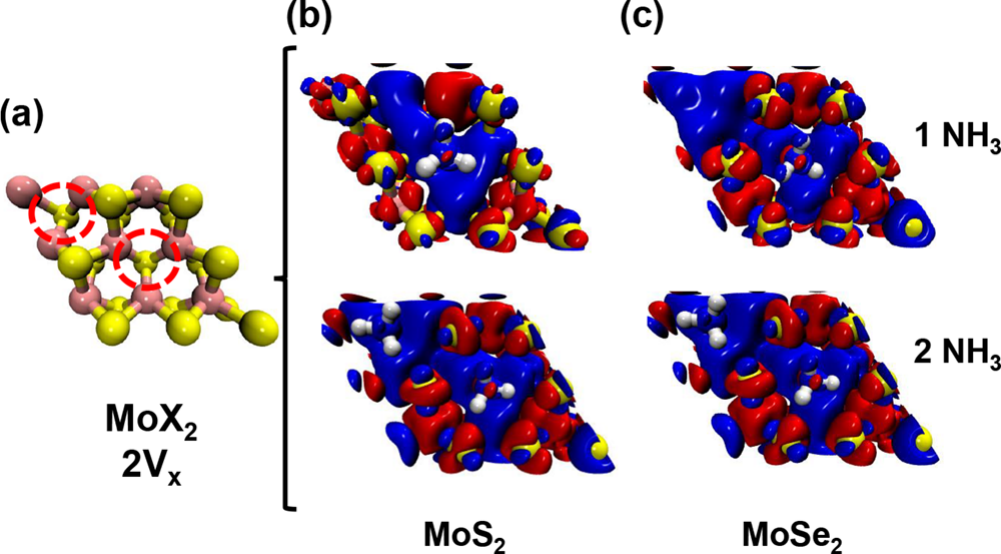
Spin density maps for (a) MoX_2_ monolayers with a divacancy, (b) MoS_2_, and (c) MoSe_2_ with one and two adsorbed NH_3_ molecules. The dashed red circle indicates the position of the vacancy. In the maps, red color indicates spin-up density, while blue represents spin-down density.

**Table 2:** Spin-Polarized Electron Distribution for Mo Atoms Near Vacancy Sites with One NH_3_ Adsorption in MoS_2_ and MoSe_2_ ^a^

	MoS_2_		MoSe_2_	
Mo atom no	μ_Local_ (1 V_S_)	μ_Local_ (2 V_S_)	μ_Local_ (1 V_Se_)	μ_Local_ (2 V_Se_)
1	–0.058	–0.133	0.065	–0.984
2	–0.222	1.158	2.098	1.265
3	–0.058	–0.232	–1.079	–0.758
4	–0.104	–0.265	0.472	2.196
5	1.418	1.287	–1.474	1.410
6	1.417	1.049	2.098	1.265
7	0.026	–0.075	–0.433	1.303
8	0.025	1.067	0.472	2.196
9	–0.332	–1.150	0.065	–0.984

a The table presents the local magnetic moments (μ_Local_) in units of μ_B_ at each Mo atom (labeled according to Figure 1), calculated as the difference in spin-up and spin-down electron populations. Notably, Mo atoms at specific positions exhibit non-zero resulting magnetic moments, with μ_Local_ values possibly indicating magnetic response. This effect intensifies with divacancy configurations, as indicated by the increased magnetic moments on other Mo atoms.

**Table 3:** Spin-Polarized Electron Distribution for Mo Atoms Near Vacancy Sites with Two NH_3_ Adsorptions in MoS_2_ and MoSe_2_ in Different Positions^a^

	MoS_2_		MoSe_2_	
Mo atom no	μ_Local_ (same side)	μ_Local_ (opposite side)	μ_Local_ (same side)	μ_Local_ (opposite side)
1	–0.058	–0.058	–0.369	0.070
2	–0.226	–0.227	1.933	2.101
3	–0.060	–0.058	–0.612	–1.089
4	–0.102	–0.104	–0.697	0.447
5	1.424	1.415	1.655	–1.481
6	1.422	1.410	1.925	2.104
7	0.024	0.031	–0.681	–0.409
8	0.027	0.032	–0.440	0.463
9	–0.340	–0.330	–0.503	0.049

a The table presents the Mo local magnetic moments (μ_Local_) in μ_B_, illustrating the resulting magnetic moment response (atom numbers labeled according to Figure 1). When the molecules were positioned on opposite sides for MoSe_2_, the μ_Local_ values resemble those from the single vacancy and one NH_3_ case (see Table 2).

We further investigated the resulting magnetic moment behavior induced by the adsorption of two NH_3_ molecules on MoS_2_ and MoSe_2_ surfaces with a single vacancy. For both MoS_2_ and MoSe_2_, placing two NH_3_ molecules on the same side of the surface resulted in a nonzero magnetic moment, which was also observed when positioning one NH_3_ molecule on each side of the surface. In the case of MoS_2_, the magnetic moment behavior was similar to that observed in configurations with a single vacancy and one NH_3_ molecule. Similarly, for MoSe_2_, placing one NH_3_ on each side of the surface produced a nonmagnetic moment response comparable to that observed in configurations with a single vacancy and one NH_3_ (see Tables 2 and 3).

We have extended our investigation to the adsorption of NH_2_ molecules on MoS_2_ and MoSe_2_ surfaces with mono and divacancies. The results, presented in Figure 4, Tables 4 and 5, reveal distinct magnetic moment behaviors compared to the NH_3_ case, providing further insights into the role of adsorbates and vacancy configurations in spin density variations.

**Figure 4:**
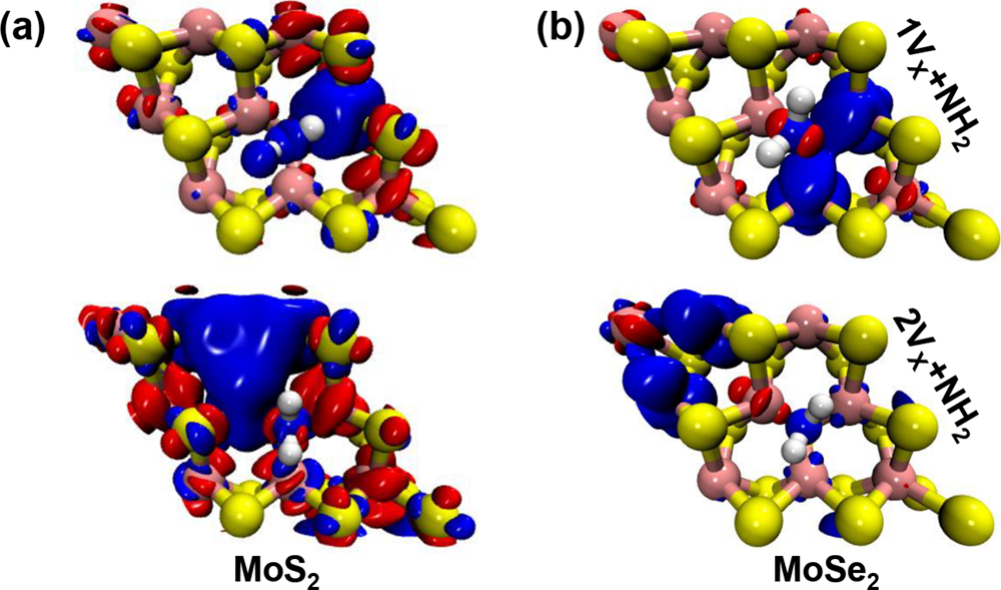
Spin density maps for (a) MoS_2_ and (b) MoSe_2_ monolayers with one and two vacancies (top and bottom figures, respectively), and with one adsorbed NH_2_ molecule.

**Table 4:** Spin-Polarized Electron Distribution for Mo Atoms Near Vacancy Sites with One NH_2_ Adsorption in MoS_2_ and MoSe_2_ ^a^

	MoS_2_		MoSe_2_	
Mo atom no	μ_Local_ (vacancy)	μ_Local_ (divacancy)	μ_Local_ (vacancy)	μ_Local_ (divacancy)
1	0.056	–0.032	0.000	0.002
2	0.032	0.692	0.041	0.020
3	–0.045	–0.051	–0.028	–0.008
4	–0.026	0.008	0.007	0.482
5	0.032	0.012	0.036	–0.050
6	0.911	0.056	0.934	0.023
7	–0.026	–0.010	0.009	–0.033
8	0.049	0.095	0.003	0.508
9	–0.044	0.188	–0.026	0.002

a The table presents the local magnetic moments (μ_Local_) in μ_B_ at each Mo atom (labeled according to Figure 1), calculated by the difference in spin-up and spin-down electron populations. Notably, Mo atoms at specific positions exhibit induced magnetic moments, with μ_Local_ values highlighting the magnetic moment response. Unlike the NH_3_ case, this effect does not increase with divacancy configurations.

**Table 5:** Spin-Polarized Electron Distribution for Mo Atoms Near Vacancy Sites with Two NH_2_ Adsorptions in MoS_2_ and MoSe_2_ ^a^

	MoS_2_		MoSe_2_	
Mo atom no	μ_Local_ (same side)	μ_Local_ (opposite side)	μ_Local_ (same side)	μ_Local_ (opposite side)
1	0.048	0.022	0.000	0.197
2	0.029	0.040	0.000	0.029
3	–0.045	–0.022	0.000	–0.036
4	–0.029	0.006	0.000	–0.008
5	0.030	0.043	0.000	0.008
6	0.910	0.825	0.000	0.973
7	–0.022	–0.007	0.000	0.032
8	0.040	0.011	0.000	–0.002
9	–0.043	–0.024	0.000	0.007

a The table presents the Mo local magnetic moments (μ_Local_) in μ_B_, illustrating the magnetic response (atom numbers labeled according to Figure 1). We found no magnetism in MoSe_2_ for the configuration with both NH_2_ molecules on the same side.

For NH_2_ adsorption, we observed a magnetic response in Mo atoms near the vacancy sites for both MoS_2_ and MoSe_2_. From the simulations, it is evident that the magnetic moment in MoS_2_ increases substantially when a divacancy is introduced; however, this behavior was not observed in MoSe_2_. In MoS_2_, significant magnetic moments appeared at specific Mo atoms, with local magnetic moments (μ_Local_) reaching up to 0.911 μ_B_ for the single vacancy, and showing only slight variations under divacancy conditions. Conversely, in MoSe_2_, the magnetic moments were generally weaker, with values below 0.5 μ_B_ in most cases (Table 4).

These findings suggest that NH_2_ adsorption alone is sufficient to induce an increase in the resulting magnetic moment of Mo atoms, but that the effect does not scale with increasing vacancy density, as observed in the NH_3_ case. This suggests that NH_2_ molecules interact differently with the MoX_2_ surface, possibly due to their smaller size and distinct electronic configuration compared to NH_3_, resulting in distinct bonding characteristics and charge redistribution near the vacancy sites. The absence of a significant enhancement in the total magnetic moment for divacancy configurations further emphasizes the localized nature of the magnetic response induced by NH_2_.

Following this, we further investigated the magnetic moment behavior induced by two NH_2_ molecules adsorbed on MoS_2_ and MoSe_2_ surfaces with a single vacancy. For MoS_2_, adsorbing two NH_2_ molecules on the surface resulted in local magnetic moments distributed among Mo atoms near the vacancy site (Table 5). For MoSe_2_, positioning one NH_2_ molecule on each side of the surface induced a magnetic moment; however, placing two NH_2_ molecules on the same side of the surface resulted in a zero magnetic moment. For clarity, Tables 2–6 report the local magnetic moments of Mo atoms near the vacancy sites, whereas the total magnetic moment of each configuration is summarized in Figure 5.

**Figure 5:**
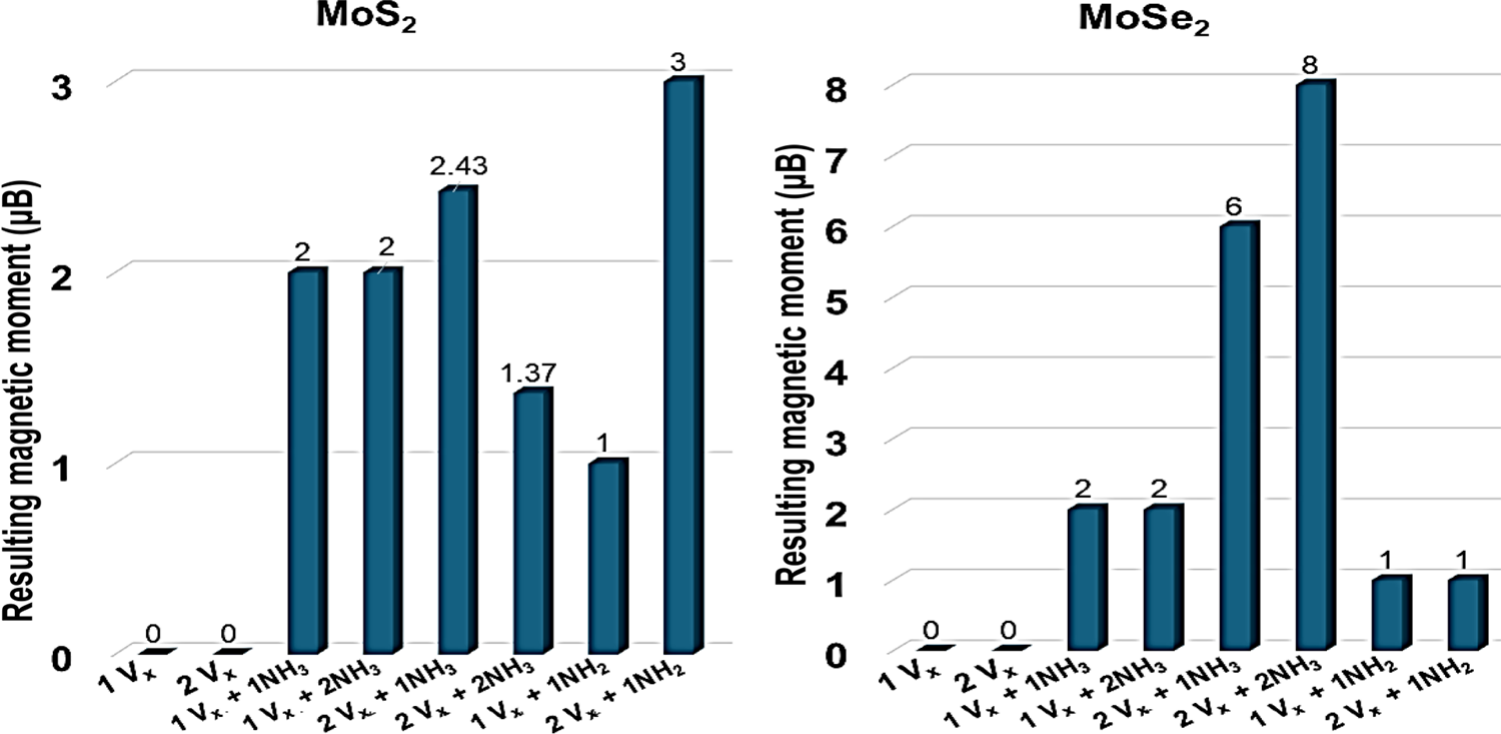
Resulting magnetic moment of each case considered in this work. The results reflect the values obtained from the same side vacancy and NH_3_/NH_2_ molecule adsorption.

**Table 6:** Spin-Polarized Electron Distribution for Mo Atoms Near Vacancy Sites with One NH_2_ and One H Adsorption in MoS_2_ and MoSe_2_ ^a^

	MoS_2_		MoSe_2_	
Mo atom no	μ_Local_ (same side)	μ_Local_ (opposite side)	μ_Local_ (same side)	μ_Local_ (opposite side)
1	–0.005	0.000	0.064	0.000
2	–0.011	–0.018	0.042	0.000
3	–0.026	–0.034	0.003	0.000
4	–0.038	–0.047	0.678	0.000
5	–0.014	–0.019	0.023	0.000
6	0.601	0.620	0.922	0.000
7	–0.038	–0.047	0.015	0.000
8	–0.041	–0.021	–0.001	0.000
9	–0.025	–0.035	–0.029	0.000

a The table presents the local magnetic moments (μ_Local_) in μ_B_ at each Mo atom (labeled according to Figure 1), calculated by the difference in spin-up and spin-down electron populations. Notably, Mo atoms at specific positions exhibit induced magnetic moments, with μ_Local_ values highlighting the magnetic moment response.

We also investigated configurations in which NH_2_ and H fragments are adsorbed on the surface, representing dissociated NH_3_ species. We considered two distinct arrangements: both fragments located on the same side of the exposed surface, and an alternating configuration in which NH_2_ and H are positioned on opposite sides (top and bottom). The corresponding results are reported in Table 6. For MoS_2_, a net magnetic moment of 0.444 μ_B_ was observed in both configurations, indicating that the dissociation induces magnetization regardless of the spatial distribution of the fragments. In contrast, for MoSe_2_, a net magnetic moment of 2.0 μ_B_ was only found when both NH_2_ and H were adsorbed on the same side of the surface, while the alternating configuration did not result in any net magnetization.

These results suggest that the magnetic response of the system is highly sensitive to both the nature of the chalcogen atom and the spatial arrangement of the dissociated species. The stronger magnetization observed in MoSe_2_ under specific adsorption geometry may be attributed to enhanced spin polarization effects mediated by the heavier selenium atoms and the localized electronic interactions between coadsorbed fragments. This highlights the potential for tuning magnetic properties in 2D materials via controlled molecular dissociation and adsorption configurations.

We extended our investigation to W-based dichalcogenides, WS_2_ and WSe_2_, using the same approach used to MoS_2_ and MoSe_2_. Initially, we explored the magnetic moment behavior of the pristine monolayers as well as systems with mono and divacancies (V_X_ and 2 V_X_, respectively). Unlike MoX_2_, no resulting magnetic moment was observed in WS_2_ or WSe_2_ for any vacancy configuration, with the magnetic moments consistently obtained as zero.

Additionally, we examined the adsorption of one and two NH_3_ molecules on the vacancy sites of WS_2_ and WSe_2_. In all cases, the systems remained with a zero resulting magnetic moment, indicating that NH_3_ adsorption does not induce spin density changes in these materials, even in the presence of vacancies. Nonzero spin density emerged only when a W atom was removed instead of an S or Se atom. This suggests that the absence of a resulting magnetic moment in the previous configurations arises from the electronic structure of the W atoms and their interaction with the surrounding lattice. This behavior is consistent with previous theoretical studies, which reported that magnetism appears only in the presence of W_2_ or WSe_6_ vacancies.^12^

Our results show that creating S or Se vacancies in MoX_2_ does not inherently lead to a nonzero resulting magnetic moment; however, the presence of NH_2_ or NH_3_ molecules can modify the local environment, leading to a nonzero magnetic moment. This effect becomes more pronounced under conditions of high defect density and low NH_3_ concentration. On the other hand, no resulting magnetic moment was observed in WX_2_ systems with either mono- or divacancies of X. These findings highlight a significant contrast between the spin density properties of Mo- and W-based dichalcogenides under similar conditions, emphasizing the critical role of the transition metal in determining the resulting magnetic moment behavior of these materials. Finally, we note that the simultaneous presence of different vacancy types, such as chalcogen and metal vacancies, is expected to introduce additional localized states and may lead to distinct magnetic responses. A reliable treatment of such mixed-defect configurations would require significantly larger supercells to avoid artificial interactions, thereby substantially increasing the computational cost. These systems, therefore, represent an interesting direction for future investigations.

## Conclusions

In summary, spin-polarized DFT simulations were employed to investigate the effects of NH_2_ and NH_3_ adsorption on defective MoX_2_ (X = S, Se) monolayers. The results confirm that pristine chalcogen vacancies do not induce magnetism, while molecular adsorption can create localized magnetic moments in Mo-based dichalcogenides. A notable case was observed for MoSe_2_, where NH_3_ dissociation into NH_2_ and H fragments on the same side of the surface produced a net magnetic moment of 2.0 μ_B_. For comparison, W-based TMDs were also examined and remained nonmagnetic under equivalent conditions. These findings suggest that molecular adsorption, combined with defect engineering, influences the magnetic behavior of Mo-based TMDs, providing insights for future studies on spin-related phenomena in low-dimensional systems.
